# Bridging the Gap: A Mixed Methods Study Investigating Caregiver Integration for People with Geriatric Syndrome

**DOI:** 10.5334/ijic.5577

**Published:** 2021-03-16

**Authors:** Isabelle Meulenbroeks, Liz Schroeder, Joanne Epp

**Affiliations:** 1 Macquarie University Centre for the Health Economy, Address: Suite 3-4, Level 1 EMC2 Building 3, Innovation Rd, Macquarie Park NSW 2109

**Keywords:** caregiver integration, geriatric syndrome, physiotherapists, acute/subacute care, care transitions, family-centred care

## Abstract

**Introduction:**

Transitions of care between acute hospital and community settings are points of vulnerability for people with geriatric syndrome. Routinely including informal caregivers into the transition processes may mitigate risk. Guidance for operational aspects of caregiver inclusion is currently lacking in healthcare policy and fails to address the barriers faced by caregivers and healthcare professionals.

**Methods:**

A questionnaire and a semi-structured interview were piloted with acute care physiotherapists who facilitate patient discharge into community settings. The questionnaire was analysed using summary statistics and interviews were thematically analysed by researchers, using NVivo 12 software.

**Results:**

Questionnaire responses indicated mixed satisfaction with current caregiver integration by the multidisciplinary team. Four themes were shaped in the interviews: inconsistent caregiver engagement, individuals working in a system, an outdated model of care, and invisible care gaps.

**Discussion:**

Feedback loops constructed from participant questionnaires and interview responses informed the identification of barriers and solutions. These are system wide and address automated integration, cultural shift, reimbursement models, and flexible structures to enhance informal caregiver participation. Future research is urgently required to translate, implement, and evaluate enhanced caregiver integration to ensure sustainable, person-centred healthcare delivery.

## Introduction

As populations age, rates of comorbidity and disability are increasing globally [1]. The concept geriatric syndrome encapsulates an elderly person’s vulnerability associated with ageing and comorbidity [2,3]. In geriatric syndrome, multiple conditions accumulate to develop non-specific multisystem impairments, for example; falls, incontinence, physical, and functional decline [4]. These have a cyclical and downward trending relationship with frailty, poor health outcomes, and eventually cause death [2].

Due to their complex care needs, persons with geriatric syndrome frequently transfer between hospital and community settings. These transitions of care are periods of risk. When transitions of care are poorly conducted, they may harm patients through medication and clinical errors [5], resulting in increased hospital readmissions [6,7] and societal cost. Person-centred care may mitigate the risks in transitions of care, as the integration of the patient, and their existing support networks may assist the development of a realistic treatment plan, and address confusion for all involved [7].

Informal caregivers are the unpaid family and friends who aid a patient, from infrequent assistance for community support to round the clock care. In Australia, 80 per cent of community care is provided by informal caregivers [8], and in 2015 it was estimated this care would have cost AU$60.3 billion a year to replace [9]. The demand for informal care is predicted to increase, due an ageing population and rise in frailty, yet the number of carers is likely to decrease, driven by changing family structures [9]. Given these contrasting trends, governments need to consider their means to maximise caregiver and health system efficiency [8,9]. Some literature suggests that including caregivers in transitions of care may decrease hospital readmissions [10–15], and improve patient and caregiver satisfaction [14,16,17,18]. Whilst a systematic review (in press) conducted by our research team established positive trends in these outcomes, the evidence is poorly established and inconclusive.

Irrespective, caregiver integration continues to receive strong support in national and international health policy documents, as a method of supporting patient-centred care [19,20,21,22]. However, in practice, it is reported as suboptimal by caregivers and healthcare professionals [23,24]. Healthcare professionals report that informal caregiver inclusion is limited by time pressures, lack of compensation or incentive, workplace culture, physical environment, lack of confidence in caregiver inclusion skills, and concerns for patient privacy and autonomy [23,25]. Current policy documents do not adequately address the barriers to caregiver inclusion faced by healthcare professionals. They also provide limited operational best practice guidance for caregiver integration.

This pilot mixed method study adopts an implementation science lens to “bridge the gap” between top-down policy recommendations and the realities experienced by healthcare professionals who provide transitional care. It explores how informal caregiver integration can be better achieved. In the past, caregiver integration literature has focused the role of nurses and case managers in this role. However, in true person-centred integrated care, discharge planning and caregiver inclusion is the responsibility of every healthcare professional [26,27]. This study conducted a pilot to explore these concepts and challenges in physiotherapists at a private hospital in New South Wales, Australia. Physiotherapists were selected because they are highly likely to encounter geriatric syndrome due to the patients’ functional decline [28]. Also, within the multidisciplinary team, their skills are essential to discharge planning and their role encompasses community social support and emphasises person centred care, and so they are also likely to interact with caregivers.

## Methods

The pilot study used a mixed method design for rich, triangulation of data. The research objectives included i) identify how physiotherapists engage caregivers during transitions of care, ii) explore what lessons could be learnt from barriers and enablers in current practice, and iii) discuss perceptions of caregiver engagement solutions by physiotherapists.

The pilot study received ethics approval from the participating facility (2019–18) and was designed and implemented in accordance with National Health and Medical Research Committee (NHMRC) guidelines. Informed consent was collected verbally at recruitment and on-paper immediately prior to participation. All collected data was deidentified using a codebook at the time of collection.

### Participants

Participants were made aware of the study through hospital department meetings and an advertising flyer. Participants were included if they were a level 1–2 physiotherapist, were working full or part time, and had experience including carers into transitions of care for patients with geriatric syndrome. They were excluded if they had not included informal caregivers in transitions of care or were a grade three physiotherapist or above. Level 1–2 physiotherapists in New South Wales practice physiotherapy in a range of sub-fields e.g., on rehabilitation, neurology, and cardiorespiratory wards. They frequently rotate between wards in a hospital setting. At the time of this study, the participating private hospital employed 26 eligible physiotherapists. The pilot was designed to recruit a minimum sample of ten physiotherapists; likely to be sufficient to reach data saturation in interviews in a homogeneous, small population [29]. To test saturation, an additional stopping criterion was applied to the study design; recruitment and interviewing would cease when no new concepts were identified in two consecutive post-interview reflections [29]. The stopping criterion was applied during the study as new concepts were journaled in reflections following the interview with participant 9, no new concepts were documented in the following two interviews.

### Study instruments

A questionnaire was designed to capture differences between practice and policy for physiotherapists providing caregiver integration, should they exist. The questions were informed by discharge guidelines [19,20,21,22,27,30,31,32] and caregiver inclusive research [10,11,12,13,14,15,16,17,33,34,35,36,37,38,39,40,41,42,43,44]. The questionnaire included an introduction and four sections which investigated: views on the quality of current practice, reflection on personal practice and knowledge, frequency of caregiver engagement by the individual and the multidisciplinary team. The questionnaire used a mixture of Likert type and 10-point Visual Analogue Scale (VAS) responses. Questions which used Likert type responses investigated frequency. There were eight possible responses ranging from never to every patient encounter (never, monthly, fortnightly, weekly, multiple times a week, daily, multiple times a day, every patient encounter). Questions using VAS responses explored participant perceptions and actions using scales that ranged from strongly agree to strongly disagree OR always to never.

The interview guide included a uniform introduction, four primary questions and a further five subset questions exploring key time points in ideal caregiver integration. Questions were designed to channel the participants’ overall experiences of caregiver integration, to reflect on their experiences, and policy expectations. The interview guide received feedback from academics in the design process and was piloted twice prior to interviewing participants. Interviews were designed to take approximately 45 minutes. The interview schedule is provided in ***Appendix 1***.

Study terms: geriatric syndrome, informal caregivers, and transitional care, were described to participants in each study instrument. In this study, geriatric syndrome was defined as patients with multimorbidity with at least one of the following symptoms: falls, pressure ulcers, incontinence, cognitive or functional decline. Participants self-determined whether they participated in discharge planning in this population. Informal caregivers were framed as any individual providing any level of unpaid support, and the term transitional care, was used interchangeably with discharge planning, and was framed as any activity which aimed to smooth the transition between settings.

Between the two study instruments we aimed to first capture the current state of caregiver integration, discharge planning, and care of people with geriatric syndrome. These results would later be contrasted and combined against in-depth narratives of experiences and perceived ideal practice to develop well-substantiated solutions to caregiver integration.

### Data collection

Participants completed the questionnaire electronically using Google Forms on a tablet provided by the researcher, then immediately following, completed a semi-structured interview. Each interview followed the primary questions laid out in the interview schedule and was conducted by the same primary researcher. Follow up questions were selected from either the interview schedule or spontaneously in response to participant experiences. The interviewer permitted silences and non-responses throughout the interview, and with caution drew on their own clinical knowledge to prompt detail. All interviews were recorded, manually transcribed verbatim, and shared amongst the team for reflection and feedback. The iterative process of interviewing was conducted at the same time as data analysis.

### Analysis

The study used a convergence model, where quantitative and qualitative data are analysed separately, then combined and contrasted in interpretation [45]. Due to the small sample size, quantitative data was analysed using summary statistics only (mean, median, and standard deviation) and trends were narratively described. Qualitative data was analysed using reflexive thematic analysis, as outlined by Braun and Clarke [46]. It followed: familiarisation with the data, generating initial codes, generating initial themes, review themes, naming and definition, and report write up. This was not conducted in a linear fashion but rather iteratively, and in some cases simultaneously [47]. The first four interviews were coded by the primary researcher and independently by two secondary coders within NVivo 12. Weekly meetings were held to discuss code development. The primary researcher maintained a detailed log of review sessions to document code refinement and development of the coding structure. The remaining seven interviews were coded independently using the agreed coding structure. A detailed memo of code and theme development, and a reflexive journal was kept by the primary researcher throughout qualitative analysis to maintain transparency [48]. All codes and themes developed were discussed and refined with the research team.

## Results

### Demographics

Physiotherapists were mostly female and middle aged. Participants, on average, selected two fields of most experience. Participants reported having the most experience in neurology (6), followed by orthopaedics, general medicine, and rehabilitation (5 each), intensive care (2), and other (3). Demographic details are provided below in ***Table 1***.

**Table 1:** Participant demographics.


CHARACTERISTIC	MEAN (RANGE)

Gender (number)	

Female	10

Male	1

Age (years)	42 (31–51)

Years of experience	18 (4–30)

Number of previous workplaces	5 (2–10)

Number of current workplaces	1.36 (1–2)


### Questionnaire results

In section one, participants described the frequency of their caregiver inclusion. Participants indicated that they saw people with geriatric syndrome multiple times a day and interacted with informal caregivers daily. When communicating about discharge planning, they most frequently discussed mobility status and mobility aids, on average this was multiple times in a week.

Section two explored perceptions of the multidisciplinary team’s frequency of caregiver engagement. Participants consistently responded that patients and caregivers were rarely included in team discussions but were likely informed of the meeting conclusions on a weekly basis. This aligned with the frequency of multidisciplinary team meetings. Participants frequently assumed that someone else in the multidisciplinary team was responsible for discharge planning and carer engagement.

Section three explored perceptions of caregiver engagement in current practice. All participants strongly agreed that caregivers should always be included in discharge planning by the multidisciplinary team and themselves (mean 9.7 and 9.4 respectively on 10-point VAS). Satisfaction regarding the quality of current caregiver integration was mixed. Six participants reported that current practice was close to their perception of ideal caregiver integration, and five disagreed.

Section four identified low levels of awareness of the policies for discharge planning and related professional codes of conduct. Despite this, participants reported they were confident in their discharge planning, but less so on when and how to include caregivers (mean 7.64 and 6.73 respectively on 10-poin VAS). Participants also strongly expressed that their physiotherapy colleagues were including caregivers as often as they deemed appropriate but had slightly higher perceptions of the multidisciplinary teams’ inclusive practices (6.45 and 7 respectively on 10-point VAS).

### Interview results

Participant interviews lasted 47.40 minutes on average and ranged from 28.25–69.58 minutes. Four themes were identified in thematic analysis, these themes are discussed below, and the coding structure is available in ***Appendix 2***.

#### Theme one: Inconsistent caregiver engagement

Early caregiver engagement was significantly enhanced by caregiver characteristics, the 3P’s: frequent caregiver **p**resence on the ward, that the caregiver was **p**roactive in seeking out healthcare professionals, and that caregiver was perceived to be **p**leasant or compliant.

“I think again it depends on the personality of of the person so in this case the wife was absolutely lovely so it was absolutely fine having her there and including them.” (participant 1, 20 years of experience)

Patient characteristics, namely poor cognition, and significant dependency in activities of daily living, were also significant facilitators for inclusive discharge practices.

Participants focused on the impact of caregiver and patient factors, and infrequently considered the impact their own traits had on caregiver inclusion, for example empathy and experience. Those who reflected on their own skills considered them essential building-blocks in the patient-caregiver-professional dynamic. However, these skills were often developed through self-initiative.

“you’ve got to be empathetic as well and uhm because that comes with experience you know and like what’s the way to say that and how do you support the caregiver it’s not something you learn uh so it’s very very hard.” (participant 9, 30 years of experience)

Social complexity emerged as a strongly complicating the initiation and maintenance of caregiver engagement, specifically convoluted family, and carer dynamics. Complex relationships can create adverse environments for caregiver engagement and hamper communication, for example, disputes over guardianship or separated families.

Altogether, the strong dependence on caregiver, patient, and individual characteristics suggests that current caregiver inclusion, in current practice, is highly variable.

#### Theme two: Individuals working in a system

Factors outside the immediate triad of the patient, caregiver, and healthcare professional had strong influence over caregiver integration. At the system level, participants identified that incentives for activity-based care were often at odds with delivering quality assured, high fidelity treatment that could involve informal caregivers.

“I’m weighing up if a patient could be seen but they are at baseline if they could be seen maybe to help potentially reduce the risk of falls in the future and give them exercise and work with the caregiver in that regard or see another patient who is not at their baseline … then I’ll see the patient who is not at the baseline yet who would benefit more at that moment.” (participant 7, 4 years of experience)

Other system level factors that impacted caregiver inclusion included participant perception of: insufficient time and staffing to cover clinical load, too few services at discharge, no communication between settings, and difficulty navigating community services.

Hospital factors impacted inclusive practices. The daily morning meetings with the multidisciplinary team enabled communication about caregiver inclusion and discharge planning but were deemed too short, generating the need for additional informal communication. Conversely, longer weekly multidisciplinary team meetings were viewed to be less productive. Detailed documentation was also essential for discharge planning and communicating inclusive caregiver practices with the team. However, the arduous time invested in capturing notes, the duplication and likely redundancy of the content captured in the electronic medical record system caused participant frustration.

“I think we are too repetitive with some of the notes and I think we don’t, I think the communication part is still the downfall the verbal stuff (informal communication).” (participant 2, 20 years of experience)

Hospital supports such as whiteboards were helpful for communication with the caregiver. However, they were seldom used and updated infrequently. Formal discussions with caregivers in family meetings were described as having a potential to be beneficial for caregiver engagement and empowerment. However, participants rarely attended the family meetings for a variety of reasons, such as time constraints, discontinuity of care and mistimed scheduling; some participants believed this was a missed opportunity.

Caregiver integration was strongly influenced by the multidisciplinary team. Informal communication within the team was a facilitator for transitions of care and caregiver engagement. Teamwork and staffing continuity improved informal communication, where long-term and familiar teams appear to have more frequent and open informal communication. Conversely, personal tensions between staff could cause infrequent and inconsistent information. A leader for discharge planning and caregiver engagement was thought to be beneficial, though there was no consistent perspective on whose job it was to lead.

#### Theme three: Outdated model of care

The care described by participants tended to be described as acute care centric and episodic. This was demonstrated by poor knowledge of community services, infrequent referrals to community services, or insufficient implementation of self-management programs e.g., falls prevention. Many participants identified that there is no incentive, from their perspective, to establish community supports and self-management.

The hospital-centric model of care also provided little clarity about where, when, or how caregivers should be included. Some assumed that including the caregivers was in lieu of patient capacity, rather than as an adjunct. For example, caregiving supports were defined specifically for impaired cognitive capacity.

Healthcare professionals’ perceptions of caregivers might also be “out of date” as they frequently inferred that the caregiver was younger and female.

“say a patient is being visited by a daughter.” (participant 6, 14 years of experience)

“the daughter of so and so wants to speak to you.” (participant 9, 30 years of experience)

“I would generally tell the patient that I’m calling the daughter.” (participant 1, 20 years of experience)

Additionally, many misinterpreted newer trends in caregiving. Namely, older spouses, co-dependency and the dynamic interactions or changes between the patient and caregiver. Some recognised these concepts but did not articulate or harness inter-dependencies for healthcare improvement. Others deferred to engaging the younger, often female, family member as opposed to the older, co-dependent partner who might live with the patient.

“Yeah so, I wouldn’t be going to the [co-dependent caregiver], because I’d be going to the daughter who lives down the road but yeah.” (participant 1, 20 years of experience)

Overall, the model of caregiving described by participants was siloed, and inconsistently facilitated person- and family-centred care, thus we described this as an outdated model of care.

#### Theme four: No solutions exist for invisible care gaps

Participants, when prompted, seemed unsure about their ability to include informal caregivers whose engagement, while crucial to care, was provided in a less intensive and visible format, for example, caregivers who did not live with patient, did not live locally to the patient, or spent their days at work. The language and lengthy pauses in participants’ responses suggests this was the first-time participants considered “invisible” caregivers.

Invisible gaps also extended to gaps in care, for example, participants assumed caregiver integration and discharge planning was being performed by someone else but, were uncertain of details. Additionally, some participants acknowledged that many patients had subtle cognitive decline or were stressed at the time of discharge. Despite this, participants, at times, described providing only verbal discharge information to patients and caregivers.

There was evidence during the interview of a progressive realisation of these care gaps, some of which were prompted or co-created by the interviewer. For example, the following co-created issue is about unintended use of the electronic medical record generated discharge letters.

“just generally speaking do you think that’s [discharge letter] written in a way that patients and caregivers could understand?” (interviewer)

“I don’t think so no.” (participant 9, 30 years of experience)

The invisibility of caregivers and care gaps hindered the participants ability to construct solutions during the interview. Of the solutions that participants did suggest, increasing individual ownership was the most common. This was the notion that healthcare workers needed to take more responsibility for their individual case load, and the person-centred care that required. Another common solution was to “give us more time” (participant 2, 20 years of experience), expressed with the view that reducing the volume of work would increase the quality of discharge planning and caregiver engagement. Participants also defaulted to responding with pre-existing models of care e.g., hiring a case manager or nurse to manage caregiver inclusion and discharge, or modules of training.

“I think that’s why a case manager was good.” (participant 2, 20 years of experience)

Other participants were uncertain of solutions. This was evidenced in their language, for example repetitions of “Umm…….” (participants 1, 4, 6, 8, 9), or repeating prompts delivered by the interviewer “Yeah education and some policy” (participant 6, 14 years of experience). Many of the solutions were co-created between the interviewer and participant.

“Do you think it’s something [empathetic caregiver engagement] people can learn to do?” (interviewer)

“Definitely.” (participant 9, 30 years of experience)

“What do you think would help them to be better at that?” (interviewer)

“I think everybody should just do some sort of training.” (participant 9)

Generally, new innovations were rarely described, either with or without assistance. Interestingly, despite their emphasis on the caregiver characteristics earlier in the interview, they did not communicate that caregivers could be part of the solution.

## Discussion

In this exploratory study we identified that physiotherapists currently engage informal caregivers in transitions of care through “professional-assistant” relationships. Here, caregivers are integrated as an additional resource, for example, being a translator, continuing to support patients with physical exercises or assisting with low intensity nursing duties. However, caution needs to be taken in these types of relationships. Caregivers are unpaid and untrained agents, not obliged to perform this care, and may experience harm as a result of caring, for example financial difficulty, poor mental health and the risk of physical injury to themselves [49,50,51]. This association with harm correlates with the intensity of caregiving, caregiver age, and may perpetuate pre-existing social inequalities such as low socioeconomic and health status [51]. Additionally, dependence on informal caregivers may not be sustainable given the burden of the ageing population, and the decreasing youth to elderly demographic (changing family structure and birth rates) [9]. Aside from the type of relationship, we also found an underlying sentiment that both caregiver engagement and overall discharge planning was not believed to be within the physiotherapists scope of practice. Future research is required to investigate multidisciplinary dynamics and roles for family-centred discharge planning.

As established in theme four, few participants provided direct solutions to caregiver engagement in discharge planning. Therefore, solutions were also constructed from barriers and enablers identified in interviews and questionnaire results (***Table 2***), and are informed by our previous research in the field [52]. ***Table 2*** summarises the barriers and enablers as perceived by the physiotherapists, at the patient, caregiver, therapist, team, facility, and healthcare system level. As such, some factors are repeated as the apply to multiple levels.

**Table 2:** Barriers and facilitators to caregiver engagement in current practice.


	BARRIER OR NOT REQUIRED	FACILITATOR

Patient	Healthy straight forward patient	Poor cognition Requires high level care Actively asks for caregivers to be involved Non-English-speaking background

Caregiver	Perceived as unrealistic by staff Older carers/spouse	Proactive Pleasant Present on the ward Culture of family caregiving

Physiotherapist	Does not feel including caregivers is worthwhile Assumes another staff member will include caregiver Differing reactions to difficult situations Time management Untrained Short shifts/part-time employment	Listening Empathy Experience Communication skills Teamwork Good documentation Contextual knowledge e.g. services

Multidisciplinary team	Personal tensions within the team Poor communication Overlapping roles Overly siloed roles Language used to describe the caregiver and patient	Teamwork Communication Strong leadership Support/comradery in the team Good documentation

Facility	Physical environment Ambiguous visitor policy Healthcare as a business Electronic medical record system design Unclear privacy policy	Structured communication for within the team Structured communication between caregivers and Communication on patient whiteboards Quick involvement of appropriate disciplines

Healthcare system	Incentivises numbers of patients treated Resource poor Work not suited to casual/part time work force Poor integration between hospital and community Hospitals staffed hours Time constraints Delayed discharges	Perception of sufficient transitional/community services Flexible working arrangements for caregivers


Healthcare professionals’ dependence on caregiver traits (theme 1 and ***Table 2***) suggests that integration could be improved by supporting caregivers to become proactive and present in-patient care. A possible solution, to facilitate caregiver proactiveness, could be to establish routine opportunities for caregiver participation at the facility. Caregiver orientated solutions are in direct contrast to participant interview responses, where none identified that caregivers could be part of the solution. Additionally, it is likely that small scale solutions at the facility level will have limited impact on overall caregiver integration as factors that influence participation may lie outside of the scope of the health system e.g., working hours or socioeconomic status. It is possible that, full caregiver integration requires a culture shift in society, to recognise the invaluable role caregivers play.

Caregiver integration is influenced by many factors beyond the immediate triad of the caregiver, patient, and clinician, such as the multidisciplinary team, facility, and health system (theme 2 and ***Table 2***). This suggests that improving caregiver integration requires multifaceted solutions to target these spheres of influence. At the system level, funding reform may be required to address the likely cause of perceived high clinical volume; emanating from Australia’s activity based funding model which remunerates activity and outputs [53]. Alternative funding models such as blended models or capitation, may shift focus from health outputs to outcomes. However, it is not known how reimbursement structures might affect informal caregiver inclusion.

Potential facility-level solutions could include creating caregiver inclusive spaces (meeting rooms, accommodation, interactive areas for rehabilitation), contact scheduling, and automation of some instruction in the electronic medical record system. Participants reported that hospital wards were at times too isolating for patients, limiting their contact with healthcare professionals or caregivers, or simultaneously too crowded hampering conversation, or creating patient privacy concerns. Additionally, consultation times are limited to traditional working hours, impacting the inclusion of working caregivers. These barriers have previously been identified in the literature [25,54]. Our participants suggested more inclusive scheduling, and logistical configuration of the wards to enhance patient and caregiver inclusion; this potential is largely unexplored in the published literature. Participants also suggested specific changes to the electronic medical record interface to capture additional content, for example structured documentation at admission to capture the presence, willingness, and availability of a caregiver. To our knowledge this has not been trialled previously. Some participants also reported using current autogenerated discharge summaries, intended for community healthcare professionals, to provide information to patients and caregivers. However, these discharge summaries are unsuitable for patients and caregivers as they contain many acronyms and are provided in an unhelpful clinically oriented format. Discharge summaries generated by the electronic medical record could be redesigned for caregiver inclusion. This idea has been trialled previously and has resulted in improvements in patient comprehension of post-discharge instructions [55]. Such changes to the electronic medical record system could pose as “back end solutions” to enable automated caregiver integration i.e., to maximise caregiver inclusion without burdening time poor healthcare professionals.

The invisibility of certain caregiver groups i.e., older caregivers, was a consistent barrier underlying themes three and four [9]. On occasion, interviewees discussed foregoing older caregiver’s integration due to their poor health. Current literature supports this perception, noting that older spousal caregivers are more likely to be pre-frail and may already experience cognitive decline [56,57]. Healthcare professionals need to navigate these relationships with caution as pre-frail does not equate to incompetence, but rather vulnerability. The complexities associated with an ageing caregiver demographic are largely unexplored, and mean healthcare professionals proceed unguided. Further research is needed to explore this evidence gap.

Through all themes, barriers, and enablers it was clear that caregiver inclusion is a nuanced and socially delicate task. The task requires healthcare professionals to navigate the complex relationship between two professional codes of conduct, respect for patient autonomy and caregiver inclusion, often without guidance or training. However, true respect for patient autonomy requires recognition of the patients’ decisions and of the complexity of decision making processes, even if the result is that the patient favours family or caregiver preferences over their own [58,59]. Achieving patient autonomy with caregiver inclusion may require development of workforce skills achieved through tailored education, enhanced communication, and conflict resolution skills.

Whilst the study focused on improving caregiver integration, there are already many factors that work well to integrate informal caregivers creating positive feedback loops. For example, flexible caregiver engagement is beneficial to patient care. Very independent patients require less support so engagement should be attuned and flexible to patient preferences. In the future, facilities may need to implement systematic, flexible structures that enable caregiver integration to include options for caregivers and for patients to opt-out. At a minimum, it would likely necessitate a handover at admission and discharge between clinical staff and the caregiver.

These proposed solutions for caregiver integration align with broader local and global policy goals for sustainable, quality healthcare, specifically New South Wales Health’s integrated care goals, the quadruple aims [27]. The quadruple aims intend to improve population outcomes and patient experience whilst simultaneously improving healthcare professionals’ satisfaction and decreasing costs to the health system [60]. Solutions proposed here; improved caregiver support, inclusion of all caregiver groups, automated caregiver integration, and funding reform to support quality care and improved health outcomes could contribute to these aims in revised policies and process guidance. Future systemic, caregiver inclusive practices may be an essential component of the complex and evolving approaches to achieving sustainable healthcare.

## Limitations

This study’s pilot design, in a single private healthcare facility, may limit the transferability of the findings. Patient demographics, staffing structures, and roles may differ from the public sector and from other private facilities. However, in Australia one third of all admissions of people aged over 65 are to private hospitals, thus investigating informal caregiver inclusion in the private sector is an essential component of systems change [61].

The questionnaire was constructed and piloted by the authors as no validated questionnaires relevant to the research questions exist. Hence, the validity of these findings is uncertain. To mitigate this, questions were informed by a detailed systematic review, and most recently, a review of global, national, and local transitional care and caregiver policies [52].

The study received a high response rate to participation, possibly due to the pre-existing relationships between the participants and interviewer. This collegial relationship is likely to have influenced the study. At times during the interview there was prompting by the interviewer, which may have also contributed to bias. However, little is known about this dynamic [62]. Furthermore, interview responses may have been influenced by prior completion of the quantitative survey, with the most likely impact that the interview captured “second thought” perceptions of caregiver integration. It is not known how accurately participants identified and recalled patients with geriatric syndrome. They possibly defaulted to talking about the most severe cases. As a result, these solutions could enhance systematic care for people on the more severe continuum of cognitive decline.

The study was piloted in physiotherapists, and the findings may not be generalisable to other members of the multidisciplinary team. Future work should consider the perspectives of other professionals participating in the multidisciplinary team.

Finally, the study may over represent fractured healthcare delivery as all participants worked part-time, and some were employed simultaneously at other facilities. However, most Australian physiotherapists are part-time employed [63,64], and for this research they reflected on their experience across a broad range of healthcare settings. Other demographic variables, such as age, gender, and years of experience with only slightly higher than national demographic for physiotherapists [63].

## Conclusion

This study helps fill a gap in current policy by identifying potential solutions to variable caregiver integration and related patient harm. We propose improvements in caregiver integration that require system change; a society-wide cultural shift to put patients and their families first, health system innovation and research into “back-end” technology, funding model redesign and a systematic approach to improving inclusive practices.

Future research is required to explore, translate, and evaluate appropriate solutions for caregiver integration. Otherwise, given the projected trends for an aging population; and with the increasing dependence on informal carers and a decreasing caregiver pool, this may become an issue of crisis.

## Appendix 1

**Table 3:** Interview schedule questions.

QUESTIONS		

1.	What is your experience of engaging informal caregivers in transitions of care?	

2.	What is an example/s of a positive experience you had whilst including caregivers?	

3.	What is an example/s of a negative experience you had whilst including informal caregivers?	

4.	Do you feel that the physiotherapists should be including informal caregivers in discharge planning/transitions of care?	

5.	In ideal practice	Do supports need to be established for successful discharge planning?

		Should informal carers be included at patient admission/preadmission?

		Should discharge planning take place during the inpatient stay?

		What actions or steps do you think are necessary for a well performed patient discharge when patients are ready to leave the facility?

		Should informal caregivers be involved in organising patient follow-up or treatment plan?

Do you have any final comments or thoughts about what we have discussed?		

## Appendix 2

**Table 4:** Coding structure.

Inconsistent caregiver engagement	Caregiver characteristics Patient characteristics Physiotherapist characteristics How the relationship between caregiver and healthcare professional forms Relationship with caregiver Social complexity

Individuals working in a complex system	Caregivers as a resource Physiotherapists perception of factors outside of their control The multidisciplinary team The hospital The healthcare system Leadership in discharge planning in the multidisciplinary team Uncertainty

Outdated model of care	Caregivers as coordinators of care Communication with caregivers Focus on care in hospital How healthcare professionals are affected by caregiver integration How caregivers are included in current practice by physiotherapists Caregiver engagement/disengagement affects the patient Perceptions of care Professionals as custodians of care

No solutions exist for invisible care gaps	Invisible problem Realisation of gaps Discharge planning involving caregivers in an ideal world What should caregiver engagement look like in an ideal world? When should caregivers be included? Flexibility Caregiver integration is necessary in a stressed system Lessons learnt from current practice Opinions on caregiver engagement Solutions perceived by physiotherapists
